# Characterization of Carboxylic Acid Reductase from *Mycobacterium phlei* Immobilized onto Seplite LX120

**DOI:** 10.3390/polym14204375

**Published:** 2022-10-17

**Authors:** Rose Syuhada Basri, Raja Noor Zaliha Raja Abd. Rahman, Nor Hafizah Ahmad Kamarudin, Wahhida Latip, Mohd Shukuri Mohamad Ali

**Affiliations:** 1 Enzyme and Microbial Technology Research Center, Faculty of Biotechnology and Biomolecular Sciences, Universiti Putra Malaysia, Serdang 43400, Selangor, Malaysia; 2 Department of Biochemistry, Faculty of Biotechnology and Biomolecular Sciences, Universiti Putra Malaysia, Serdang 43400, Selangor, Malaysia; 3 Department of Microbiology, Faculty of Biotechnology and Biomolecular Sciences, Universiti Putra Malaysia, Serdang 43400, Selangor, Malaysia; 4 Centre of Foundation Studies for Agricultural Science, Universiti Putra Malaysia, Serdang 43400, Selangor, Malaysia

**Keywords:** carboxylic acid reductase, *Mycobacterium phlei*, characterization, immobilization, stability

## Abstract

A multi-domain oxidoreductase, carboxylic acid reductase (CAR), can catalyze the one-step reduction of carboxylic acid to aldehyde. This study aimed to immobilize bacterial CAR from a moderate thermophile *Mycobacterium phlei* (*Mp*CAR). It was the first work reported on immobilizing bacterial CAR onto a polymeric support, Seplite LX120, via simple adsorption. Immobilization time and protein load were optimized for *Mp*CAR immobilization. The immobilized *Mp*CAR showed optimal activity at 60 °C and pH 9. It was stable over a wide range of temperatures (10 to 100 °C) and pHs (4–11), retaining more than 50% of its activity. The immobilized *Mp*CAR also showed stability in polar solvents. The adsorption of *Mp*CAR onto the support was confirmed by Scanning Electron Microscopy (SEM), Fourier-Transform Infrared (FTIR) spectroscopy, and Brunauer–Emmett–Teller (BET) analysis. The immobilized *Mp*CAR could be stored for up to 6 weeks at 4 °C and 3 weeks at 25 °C. Immobilized *Mp*CAR showed great operational stability, as 59.68% of its activity was preserved after 10 assay cycles. The immobilized *Mp*CAR could also convert approximately 2.6 mM of benzoic acid to benzaldehyde at 60 °C. The successfully immobilized *Mp*CAR on Seplite LX120 exhibited improved properties that benefit green industrial processes.

## 1. Introduction

Over the decades, many efforts have been devoted to researching potentially robust aldehyde-producing enzymes. Aldehydes are organic compounds in the fine chemicals, pharmaceuticals, and flavor and fragrance industries. They are essential intermediates for preparing high-value-added compounds, such as alkanes, alcohols, and amines. Carboxylic acids are desirable precursors for aldehyde production as they are abundant, stable, and usually biologically synthesized [1,2,3,4]. Carboxylic acid reductase (CAR) is a large (~130 kDa) multi-domain enzyme that can catalyze the one-step reduction of carboxylic acids to corresponding aldehydes with the availability of cofactors; adenosine triphosphate (ATP) and nicotinamide adenine dinucleotide phosphate (NADPH), and this enzyme demands post-translational modification for its activation [5].

CAR (EC 1.2.1.30) belongs to the aldehyde oxidoreductase group, where its first gene, *car*, was cloned, expressed, and characterized from *Nocardia* sp. [6]. The structure of CAR is relatively complex. It consists of an N-terminal adenylation domain (A domain), a thiolation domain (T domain), and a C-terminal reductase domain (R domain). Generally, the irreversible reduction of carboxylic acids to aldehydes involves three key steps: (i) Adenylation: ATP-dependent activation of the acid by the A domain, which results in the formation of an acyl adenylate intermediate; (ii) Thiolation: transfer of the acyl intermediate to the phosphopantetheine linker; and (iii) Reduction: the NADPH-dependent reduction of the acyl-thioester produces the corresponding aldehyde product [5]. For the enzyme to be in the holo form (active), a phosphopantetheine group (also known as ‘swinging arm’) needs to be attached to the conserved serine in the T domain. A phosphopantetheine transferase enzyme (PPTase) is required for CAR activity to be maximal. PPTase helps in the covalent attachment of the phosphopantetheine group [7]. The most co-expressed PPTase was the surfactin phosphopantetheinyl transferase (Sfp) from *Bacillus subtilis* [8,9,10,11,12,13].

Enzyme usage for industrial applications and commercialization purposes is broadly recognized, yet their stability and cost are treated as a limitation. The structural stability of some enzymes is highly challenged during biochemical reactions. In addition to eliminating those obstacles, enzyme immobilization is a promising approach for obtaining superior biocatalysts. Immobilized enzymes are physically confined or localized in a defined space region with retention of their catalytic activities, which can be used repeatedly and continuously [14]. A few established immobilization methods include physical adsorption, cross-linking, covalent bonding, encapsulation, and entrapment [15,16]. Each method has its advantages and disadvantages and may contribute to significant variations in the properties of the immobilized enzymes. In general, enzyme immobilization is limited by low binding on the support, lack of biocompatibility, and commonly lower residual activity than free enzyme. Some recent immobilization methods, such as metal-protein hybrids and enzyme immobilization using biocompatible supports, retain higher residual activity [17,18]. Among all the methods, physical adsorption is the simplest technique for enzyme immobilization. It depends on the enzyme-support’s van der Waals forces, hydrogen bonds, and ionic bonding. Via this method, no chemical bonding is involved between the support and enzymes, and therefore no or less significant changes to the enzyme structure [19]. Enzymes immobilized via the adsorption method have demonstrated improved properties such as retaining higher catalytic efficiency and residual activity, thermal and pH tolerance, storage stability, and recyclability [20,21,22,23].

Another important prerequisite in enzyme immobilization is the selection of suitable carriers or supports. Immobilization on ionic exchange resins is generally known to be simpler than on other supports. There are ionic and electrostatic interactions involved between the protein and the oppositely charged resin. Cationic exchangers have cations as their active ions, while anionic exchangers have anions. The enzyme is only attached to the support when there are high enough ionic bridges formed between the protein and the support to counterbalance the ionic strength of the surrounding medium [24]. This strategy minimizes the enzyme chemical modification to only the protein groups involved during the immobilization process [25]. CAR’s catalytic activity involves all three domains and occurs sequentially from A- to T- and later to R-domain, which requires high flexibility and mobility. Therefore, immobilization using ionic resins is considered suitable for CAR since the attachment of the enzyme onto the support is only at a certain region. This method will not totally ‘immobilize’ the whole CAR protein structure. Other advantages of utilizing the ionic exchange resin concept include support recovery, non-expensive, and high availability of resins. Commonly, ion exchange resins are made of polymers. Many polymeric resins have been used for many enzymes. Polymers can protect biomolecules from denaturation, inactivation, and structural damage while maintaining high catalytic activity [15].

Carboxylic acid reductase from *Mycobacterium phlei* (*Mp*CAR) was initially expressed and characterized by Finnigan et al. [26]. The enzyme is among the most thermostable CARs known to date, as it can retain 92% of its catalytic activity at 42 °C and has a residual activity of up to 50 °C. Moreover, *Mp*CAR showed the longest half-life, 123.2 h at 30 °C [26]. This enzyme showed a great pH tolerance of >50% activity between pH 4.3 and 11.8 [27]. Due to the thermostability of the enzyme, *Mp*CAR has been involved in an in vitro enzyme cascade reaction developed using a mathematical model with other enzymes, including esterase and aldehyde dehydrogenase, for the generation of 4-methylbenzyl alcohol [28]. Additionally, it is still early in the day for the immobilization of CARs. CAR immobilization was first tried using EziG Opal, a commercial support with 26% enzyme weight on the carrier and more than 59% activity recovery [29]. Recently, a CAR from *Pycnoporus cinnabarinus* fungus was immobilized onto nickel sepharose resin and achieved 82% and 76% of immobilization yield and efficiency, respectively [30].

In an attempt to create diversity in the toolbox of available CARs that may contribute to the development of sustainable and green chemistry routes, in this research, a carboxylic acid reductase from a moderate thermophile, *Mycobacterium phlei* (*Mp*CAR), was immobilized onto the commercial support Seplite LX120 via an adsorption method. This new polymeric support is an ionic resin that is supposed to provide gentle binding for the enzyme and allow the structure to maintain its flexibility, besides taking advantage of the simplicity of the immobilization process. The immobilization conditions were optimized by considering the immobilization time and protein load. The successful immobilization of *Mp*CAR was confirmed by enzyme activity assay, Brunauer–Emmett–Teller (BET) analysis, and Fourier-Transform Infrared (FTIR) spectroscopy analysis. In addition, the properties of the immobilized enzyme at different temperatures and pHs were also explored. The morphology of the support before and after CAR binding was characterized by using Scanning Electron Microscopy (SEM). More importantly, the storage stability, reusability, bioconversion ability, and the effect of organic solvents on immobilized *Mp*CAR were all investigated to determine the potential industrial applicability of this enzyme.

## 2. Materials and Methods

### 2.1. Chemicals, Reagents, and Equipment

All chemicals and equipment used in this study were obtained from the Enzyme and Microbial Technology (EMTech) Research Center, Faculty of Biotechnology and Biomolecular Sciences, Universiti Putra Malaysia.

### 2.2. Preparation of Purified Recombinant MpCAR

The gene sequence of *Mp*CAR was extracted from the NCBI database (WP_003889896.1) and was codon optimized for expression in *Escherichia coli*. The gene was synthesized by Integrated DNA Technologies (IDT). The *Mp*CAR gene was cloned into pET51b and transformed into *E. coli* BL21 (DE3). *Mp*CAR was co-transformed with a pET28a vector containing a phosphopantetheine transferase from *Anoxybacillus geothermalis* strain D9. Cells were cultured at 25 °C and expressed using 0.75 mM of isopropyl β-d-1-thiogalactopyranoside (IPTG) in Luria Bertani media for 20 h. The cells were harvested through centrifugation at 10,000 rpm for 30 min at 4 °C. The cells were then resuspended with a buffer (20 mM HEPES pH 7.4 containing 20 mM of imidazole and 500 mM of NaCl), lysed by sonication, and centrifuged again at 10,000 rpm for 30 min to collect the soluble proteins. A one-step purification was done by using nickel affinity chromatography. The column was equilibrated with a binding buffer (20 mM HEPES pH 7.4 containing 20 mM of imidazole and 500 mM of NaCl) and then loaded with the crude enzyme. The column was washed with a washing buffer for up to 5 column volumes. The purified protein underwent gradient elution using an elution buffer (20 mM HEPES pH 7.4 containing 500 mM of imidazole and 500 mM of NaCl). The eluted purified protein from each fraction was then measured for protein content using the Bradford assay and the enzyme activity assay at 340 nm (as mentioned in Section 2.3 and Section 2.4, respectively). The purified enzymes were stored at 4 °C.

### 2.3. Protein Content Determination

The Bradford assay was used to determine the protein concentration [31]. It was conducted using the commercial Bradford reagent from Sigma. The absorbance was measured at 595 nm. Bovine serum albumin (BSA) was used as the protein standard.

### 2.4. Enzyme Activity Assay of MpCAR

The assay was modified from the previously published method [32]. It was conducted in 100 mM of HEPES pH 7.5, 1 mM of ATP, 0.25 mM of NADPH, 10 mM of MgCl_2_, 10 µL (0.5 mg/mL) of purified *Mp*CAR solution, or 10 mg of immobilized *Mp*CAR (7.5 mg *Mp*CAR/g of Seplite LX120 (after optimization)), and 5 mM of benzoic acid as substrate, in a total reaction volume of 200 µL. The assay was performed in triplicate at the optimum temperature (40 °C for the free enzyme and 60 °C for the immobilized enzyme) for 10 min of incubation. The assay for free *Mp*CAR was conducted in a 96-well microplate. In contrast, for immobilized *Mp*CAR, the 10 min assay incubation was conducted in a round-bottom 2 mL microcentrifuge tube before the supernatant was transferred to the 96-well microplate for an absorbance reading. The NADPH oxidation was measured at 340 nm. Control reactions were performed by incubating all the assay components without the presence of the enzyme. One unit of CAR activity was defined as the rate of 1 µmole of NADPH consumed per minute. The enzyme activity and relative activity were calculated as below:

$$\mathrm{Free}\;\mathrm{enzyme}\;\mathrm{activity}\;(U/\mathrm{mL})=\frac{\left(\mathrm{Absorbance}\;\mathrm{of}\;\mathrm{control}-\mathrm{Absorbance}\;\mathrm{of}\;\mathrm{sample}\right)÷\mathrm{Gradient}\;\mathrm{of}\;\mathrm{NADPH}\;\mathrm{curve}}{\mathrm{Incubation}\;\mathrm{time}\times \mathrm{Volume}\;\mathrm{of}\;\mathrm{free}\;\mathrm{enzyme}}$$

$$\mathrm{Immobilized}\;\mathrm{enzyme}\;\mathrm{activity}\;(U/g)=\frac{\left(\mathrm{Absorbance}\;\mathrm{of}\;\mathrm{control}-\mathrm{Absorbance}\;\mathrm{of}\;\mathrm{sample}\right)÷\mathrm{Gradient}\;\mathrm{of}\;\mathrm{NADPH}\;\mathrm{curve}}{\mathrm{Incubation}\;\mathrm{time}\times \mathrm{Weight}\;\mathrm{of}\;\mathrm{immobilized}\;\mathrm{enzyme}}$$

$$\mathrm{Relative}\;\mathrm{activity}\;(\%)=\frac{\mathrm{Enzyme}\;\mathrm{activity}}{\mathrm{Initial}\;\mathrm{enzyme}\;\mathrm{activity}}\times 100$$

### 2.5. Immobilization of Purified MpCAR

The immobilization optimization was performed to determine the optimal conditions for the immobilization of *Mp*CAR. The optimization included the immobilization time and the protein load. The immobilized enzyme activity and the immobilization yield were recorded.

#### 2.5.1. Effect of Immobilization Time of *Mp*CAR

The effect of time on the immobilization of *Mp*CAR was conducted by mixing enzyme solution with Seplite LX120 (5 mg of protein/g of Seplite LX120) in 20 mM of HEPES pH 7.5 buffer and stirring over different periods (30, 60, 90, 120, and 150 min) at 25 °C in separate beakers. The mixture was stirred at 250 rpm. The mixture was then filtered and dried at 30 °C for 90 min in a fluid bed dryer. The unbound enzyme was measured for protein content. The immobilized *Mp*CAR was assayed for its enzyme activity. The immobilization yield was calculated as below:

$$\mathrm{Immobilization}\;\mathrm{yield}\;(\%)=\frac{\left(\mathrm{Initial}\;\mathrm{protein}\;\mathrm{content}-\mathrm{Unbound}\;\mathrm{protein}\;\mathrm{content}\right)}{\mathrm{Initial}\;\mathrm{protein}\;\mathrm{content}}\times 100$$

#### 2.5.2. Effect of Immobilization Protein Load of *Mp*CAR

The determination of the optimal enzyme concentration to be loaded onto the support was completed by varying the enzyme concentrations (2.5, 5.0, 7.5, 10.0, 12.5, and 15.0 mg of *Mp*CAR/g of Seplite LX120). Each mixture was incubated for 90 min (based on the optimum adsorption time determined earlier) at 25 °C and stirred at 250 rpm. The mixture was then filtered and dried at 30 °C for 90 min. The unbound enzyme was measured for protein content. The immobilized *Mp*CAR was assayed for its enzyme activity. The immobilization yield was also calculated using the equation shown in Section 2.5.1.

### 2.6. Characterization of the Immobilized and Free MpCAR

#### 2.6.1. Effect of Temperature

The effect of temperature on the catalytic activity of immobilized *Mp*CAR was measured at temperatures ranging from 20 to 80 °C, at 10 °C intervals, for 10 min. It was then assayed spectrophotometrically. The temperature stability of the immobilized *Mp*CAR was tested by preincubating the immobilized *Mp*CAR at different temperatures (10 to 100 °C, with 10 °C intervals) for 30 min before being assayed at 60 °C for 10 min. The control used for this experiment was the untreated enzyme.

#### 2.6.2. Effect of pH

The effect of pH on immobilized *Mp*CAR activity was evaluated at pH 4–11, by using 50 mM sodium acetate (pH 4–6), 50 mM sodium phosphate (pH 6–8), 50 mM Tris HCl (pH 8–9), and 50 mM glycine NaOH (pH 9–11). The activity assay using these different buffers was performed at 60 °C for 10 min. Moreover, the pH stability of the immobilized *Mp*CAR was determined by preincubating the 10 mg immobilized *Mp*CAR at different pH values ranging from pH 4–11 at 50 °C for 30 min. The mixture was then subjected to the activity assay at 60 °C for 10 min.

#### 2.6.3. Effect of Organic Solvents

A stability study of the immobilized *Mp*CAR towards different organic solvents was conducted. Ten mg of immobilized *Mp*CAR were mixed with 100 mM HEPES buffer pH 7.5 and 25% (*v/v*) organic solvents before preincubating for 30 min at 50 °C. The preincubated immobilized enzyme was then assayed at 60 °C for 10 min for enzyme activity. A control reaction (all assay components including the respective solvent but without the presence of enzyme) was prepared for each set of enzyme and organic solvent. The same procedure was applied to the free enzyme, except the preincubation was done at 30 °C for 30 min and the assay was performed at 40 °C for 10 min. The untreated enzyme was assigned a value of 100% activity.

#### 2.6.4. Scanning Electron Microscopy (SEM) and Brunauer–Emmett–Teller (BET) Analysis

The morphology, or surface features, of immobilized *Mp*CAR were viewed via SEM. The sample was coated with gold before being analyzed under SEM. The images of the empty support and the immobilized *Mp*CAR were captured under 20×, 500×, 5000×, and 10,000× magnifications. The surface area and pore characteristics of the immobilized *Mp*CAR were determined by BET analysis with the nitrogen gas adsorption-desorption method using the MicroActive TriStar II Plus 2.03 surface area analyzer.

#### 2.6.5. Fourier-Transform Infrared (FTIR) Spectroscopy

Structural analysis was conducted using FTIR spectroscopy. The measurement range was carried out at a spectrum range of 4000–500 cm^−1^ and over 3 accumulation scans. The spectrometer radiation (IR) was from an attenuated total reflectance (ATR) crystal. A pressure controller was adjusted for optimal contact between the sample and the diamond plate before the measurements were recorded.

#### 2.6.6. Storage Stability and Reusability Study

The storage stability of immobilized and free *Mp*CAR was determined by measuring the enzyme activity after weeks of storage. The dry powder of immobilized *Mp*CAR (7.5 mg *Mp*CAR/g of Seplite LX120) and the free purified *Mp*CAR (5 mg of *Mp*CAR in 20 mM of HEPES pH 7.5) were stored at 4 and 25 °C, respectively. For the reusability test, 10 mg of immobilized *Mp*CAR was weighed, placed in a 2 mL microcentrifuge tube, and prepared for the enzyme activity assay at pH 7.5. After each assay cycle, the mixture of immobilized *Mp*CAR and other assay components was centrifuged to separate the supernatant from the immobilized enzyme (the pellet). The supernatant was then measured spectrophotometrically at 340 nm. The immobilized enzyme (pellet) was washed with buffer and allowed to air dry. This process was repeated ten times. The initial activity of the immobilized enzyme was calculated as being 100%.

### 2.7. Bioconversion Analysis of Immobilized MpCAR Using High-Performance Liquid Chromatography (HPLC)

The enzyme (10 mg of immobilized *Mp*CAR (7.5 mg *Mp*CAR/g of Seplite LX120)) was incubated with other assay components, as mentioned in Section 2.4. In separate microcentrifuge tubes, the reaction mixture was shaken moderately and incubated for 1 h at different incubation temperatures ranging from 20 to 60 °C (with 10 °C intervals). The supernatant was transferred to HPLC vials for analysis. Two individual experiments were performed, and the conversions of benzoic acid to benzaldehyde were quantified using HPLC-UV. HPLC-UV measurements were conducted, as explained in [33]. Benzaldehyde was detected at 254 nm. Product quantification was calculated using linear interpolation from the benzoic acid calibration curve. The bioconversion yield was calculated as below:

$$\mathrm{Bioconversion}\;\mathrm{yield}\;(\%)=\frac{\mathrm{Concentration}\;\mathrm{of}\;\mathrm{product}}{\mathrm{Concentration}\;\mathrm{of}\;\mathrm{substrate}}\times 100$$

## 3. Results and Discussion

### 3.1. Immobilization of MpCAR

**Figure 1:**
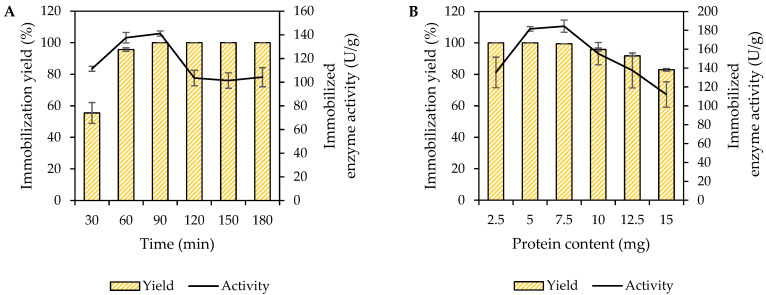
Immobilization optimization of carboxylic acid reductase from *Mycobacterium phlei* (*Mp*CAR). (**A**) Effect of immobilization time on immobilization yield (%) and immobilized enzyme activity (U/g). The purified *Mp*CAR (5 mg) was immobilized onto 1 g of Seplite LX120 at different immobilization times at 30 min intervals. (**B**) Effect of protein load on immobilization yield (%) and immobilized enzyme activity (U/g). Different protein contents (with 2.5 mg intervals) were loaded for their 90 min of immobilization onto Seplite LX120. The immobilization was performed at room temperature with a stirring speed of 250 rpm. Samples were measured in triplicates.

Seplite LX120 is a highly cross-linked styrene-divinylbenzene copolymer (containing an amine functional group) used as the immobilization support for *Mp*CAR. The immobilization of *Mp*CAR onto Seplite LX120 was optimized by varying the immobilization time to determine the optimum time with the highest protein yield and immobilized enzyme activity. Five mg of protein/g of Seplite LX120 was used as the standard concentration for this parameter. Figure 1A represents a chart with immobilization yield (%) versus the immobilized enzyme activity (U/g) at different immobilization times, with 30 min intervals. Overall, the immobilization yield of the *Mp*CAR gradually increased over time. *Mp*CAR reached its 100% adsorption yield onto 1 g of Seplite LX120 support at 90 min of immobilization time. The activity of the immobilized enzyme was also found to be the highest (141.14 U/g) at 90 min into the immobilization process. Interestingly, the 100% immobilization yield was constant even at a longer period of immobilization, up to 180 min. Perhaps the enzyme-support affinity was good; hence, the enzymes remained attached to the support. However, even though the yield was high, the activity of immobilized enzymes was observed to drop after 90 min of immobilization. It was probably due to the excessive enzyme-support interaction time that offered multi-point binding and unnecessary structural rigidity, leading to enzyme inactivation [34]. Therefore, the optimal immobilization time for *Mp*CAR onto Seplite LX120 was 90 min at 25 °C (room temperature).

Figure 1B depicts the effect of different protein concentrations loaded for the *Mp*CAR immobilization onto 1 g of Seplite LX120 for 90 min of immobilization time (based on the previously optimized parameter). At the concentration of 2.5–7.5 mg of enzyme, approximately 99–100% of the immobilization yield was obtained. However, when the protein load was further increased to 10, 12.5, and 15 mg, the yield dropped to 95, 91, and 82%, respectively. Consequently, when 15 mg of protein was used, the activity of the immobilized enzyme decreased significantly to 112.06 U/g. Similar behavior was observed when optimizing the immobilization condition of puerarin glycosidase from *Microbacterium oxydans* CGMCC 1788 onto DEAE-52 cellulose [35]. The increase in protein load permits a greater available enzyme amount to interact with the support, increasing the support surface coating. However, a higher *Mp*CAR load also causes the immobilization yield to decrease. The support may possibly have been fully coated by the *Mp*CAR and reached its saturation limit when the amount of enzyme was ≥10 mg; hence the protein is prone to leach out from the support, causing yield reduction. Considering both immobilization yield and activity of the enzyme, the optimal concentration for protein loading was 7.5 mg *Mp*CAR/g of Seplite LX120. Under these conditions, 99% yield and 184.4 U/g of immobilized *Mp*CAR activity were achieved.

**Figure 2:**
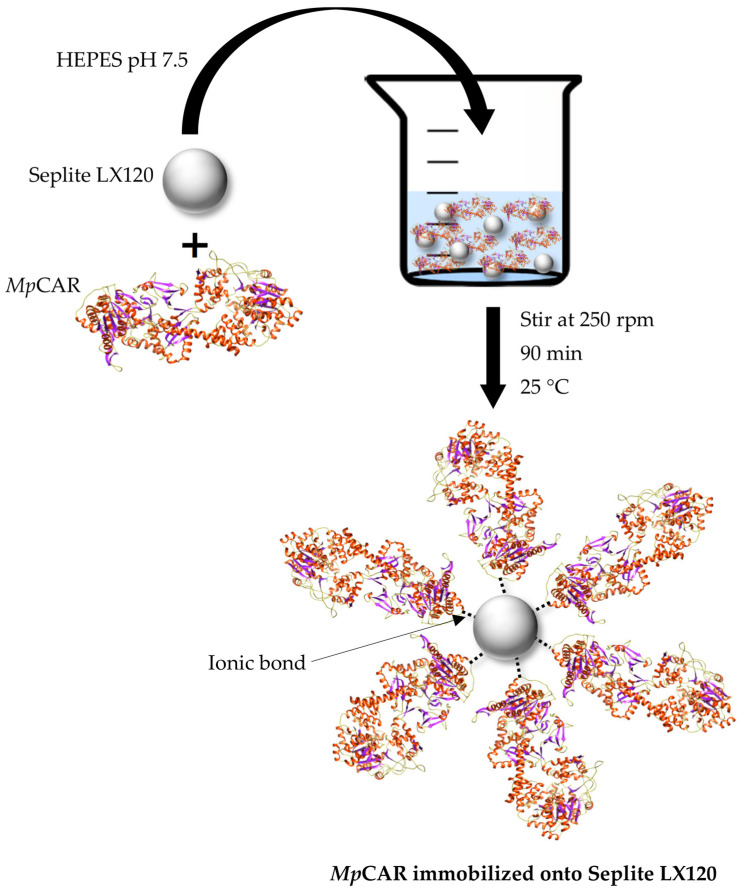
Schematic diagram illustrating the immobilization of carboxylic acid reductase from *Mycobacterium phlei* (*Mp*CAR) onto Seplite LX120 via the adsorption method.

Ultimately, based on the optimization study, the best immobilization conditions were 1 g of Seplite LX120 as the support for 90 min immobilization of 7.5 mg of purified *Mp*CAR. The stirring was maintained at 250 rpm, and the immobilization of *Mp*CAR was conducted at 25 °C (room temperature). The schematic illustration of *Mp*CAR immobilization and binding of the enzyme onto the support is presented in Figure 2.

### 3.2. Effect of Temperature on Activity and Stability of Immobilized MpCAR

**Figure 3:**
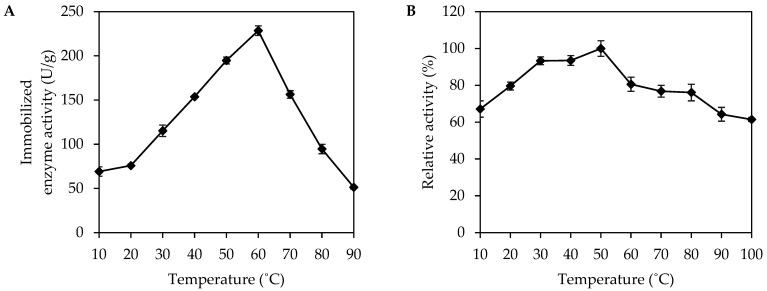
Characterization of immobilized carboxylic acid reductase from *Mycobacterium phlei* (*Mp*CAR). The effect of temperature on the activity (**A**) and stability (**B**) of the immobilized *Mp*CAR. The optimal temperature and thermal stability of immobilized *Mp*CAR were measured at different temperatures ranging from 10 to 90 °C and from 10 to 100 °C, respectively. Samples were measured in triplicates.

The temperature dependence of the enzyme activity of immobilized *Mp*CAR was studied at 10–90 °C. As shown in Figure 3A, the highest immobilized enzyme activity, equivalent to the optimal reaction temperature for immobilized *Mp*CAR, was at 60 °C. The enzyme activity of the immobilized *Mp*CAR rapidly decreased above 60 °C. A previous study showed free *Mp*CAR to possess optimal activity at 42 °C [26]. The immobilized *Mp*CAR exhibited an 18 °C increase in temperature optima compared to the free *Mp*CAR. These data suggest that immobilization increased the resilience of the enzyme, making the immobilized *Mp*CAR have better thermal tolerance than the free form. These results could be attributed to the interaction of the enzyme and support, which might impair conformational flexibility, requiring a higher temperature for the enzyme molecule to attain a proper conformation to maintain its reactivity. Hence, a sharp loss in activity above 60 °C might be due to the denaturation of enzyme molecules [36]. A similar result was obtained when an oxidoreductase laccase was immobilized and showed a higher optimal temperature (65 °C) than in its free form (55 °C). The interaction between the laccase and its immobilization support increased the activation energy to recognize the optimal conformation for substrate binding [37].

The thermal stability of immobilized *Mp*CAR was determined by measuring the relative activity as a function of temperature in the range from 10 to 100 °C (Figure 3B). It was previously discovered that free *Mp*CAR could retain its residual activity at temperatures as high as 50 °C [26]. In this study, the immobilized enzyme retained more than 60% of its relative activity across all temperatures tested, and the immobilized enzyme was found to be most stable at 50 °C. The immobilization method may have improved the conformational stability of the *Mp*CAR enzyme in its native form. The Seplite LX120 may protect the enzyme by decreasing enzyme mobility and thermal vibrations, preventing unfolding and enzyme aggregation. It is usually found that an immobilized enzyme has higher thermal stability than a free enzyme due to the restriction of the enzyme’s conformational flexibility. The enzyme became less flexible, possibly due to the attachment of the enzyme onto the support, which limits the conformational alterations and movements under different temperatures [38]. Therefore, the described immobilization process produced immobilized *Mp*CAR with excellent thermal stability.

### 3.3. Effect of pH on Activity and Stability of Immobilized MpCAR

**Figure 4:**
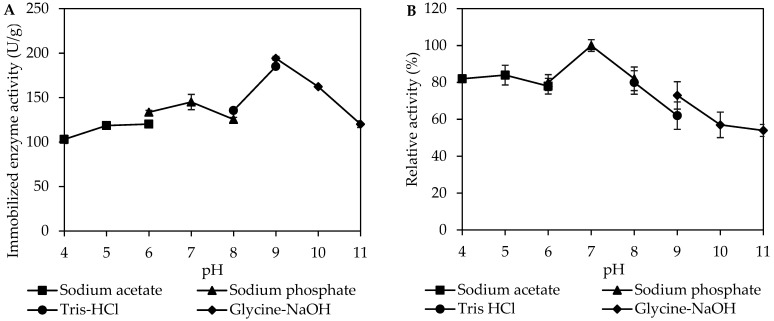
Characterization of immobilized carboxylic acid reductase from *Mycobacterium phlei* (*Mp*CAR). The effect of pH on the activity (**A**) and stability (**B**) of the immobilized *Mp*CAR. The optimal pH and pH stability of immobilized *Mp*CAR were measured at different pH ranges from pH 4 to 11. Samples were measured in triplicates.

The effect of pH on the immobilized enzyme activity depends on the enzyme, immobilization method, and support used. The effect of pH (4–11) on the activity of the immobilized *Mp*CAR was studied. As shown in Figure 4A, the optimal pH corresponding to the highest activity of immobilized *Mp*CAR was obtained at pH 9. In comparison, the free *Mp*CAR showed optimal activity at pH 7.5 and decreased quickly as the pH increased [26]. The immobilization did cause a shift in the optimal pH for the activity of immobilized *Mp*CAR, and the enzyme activity was also higher at high pH values, suggesting that the immobilized enzyme had improved alkaline resistance. The pH shifts upon immobilization probably occur due to secondary interactions between the enzyme and the polymeric support [36,39]. It may also be suggested that the Seplite LX120 is an anionic support as the enzyme immobilization causes a shift to the basic pH values [40]. The shift in optimal pH of the immobilized enzyme through simple adsorption has also been observed when pectinase was immobilized onto a cationic polystyrene resin [41].

The pH of the reaction highly influences the catalytic stability of enzymes. The influence of pH on the stability of immobilized *Mp*CAR is shown in Figure 4B. The *Mp*CAR immobilized onto Seplite LX120 remained stable (with ~50% retention in activity) within the entire pH range tested, from pH 4 to 11. In the pH range from 4 to 8, more than 80% of the relative enzymatic activity was retained, with 100% of *Mp*CAR activity achieved at pH 7. While from pH 9–11, at least >50% of enzymatic activity was obtained, with the lowest value observed being 54% at pH 11. The free *Mp*CAR was also found to have great pH tolerance, ranging from pH 4.3 to 11.8 [27]. This result showed that the immobilization procedure could maintain the stability of *Mp*CAR over a broad pH range under extreme acidic and alkaline pHs. It was confirmed that the surrounding pH was responsible for the enzyme activity as it affected the ionization within the enzyme. Most probably, the immobilized *Mp*CAR showed a strong affinity towards the substrate due to the orientation and readily available active sites of the enzyme [42,43].

### 3.4. Effect of Organic Solvents on the Stability of Immobilized MpCAR

**Figure 5:**
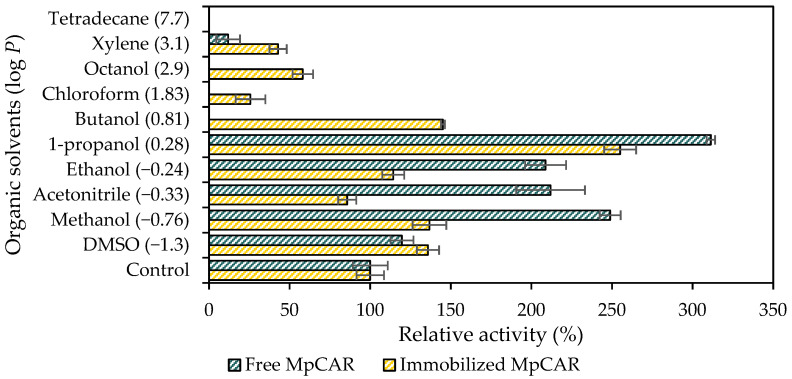
Effect of organic solvents on the stability of free and immobilized carboxylic acid reductase from *Mycobacterium phlei* (*Mp*CAR). Green columns: relative activity of free *Mp*CAR; yellow columns: relative activity of immobilized *Mp*CAR onto Seplite LX120. Log *p* is the partition coefficient of the solvent between water and octanol. Samples were measured in triplicates.

The effect of 25% (*v/v*) organic solvents on the stability of free and immobilized *Mp*CAR is shown in Figure 5. This study is intended to explore the potential of *Mp*CAR for industrial applications, as organic solvents are commonly used to increase substrate solubility and suppress water-dependent side reactions [44]. The free and immobilized *Mp*CAR showed stability in 25% (*v/v*) of most hydrophilic, polar solvents (log *p* ≤ 1), as proven by their relative catalytic activities, which retained >50% (Figure 5). In contrast, when treated with organic solvents with log *p* ≥ 1 (non-polar or hydrophobic organic solvents), the free *Mp*CAR showed a deleterious effect in their catalytic activities compared to the control. It was possibly due to the presence of organic solvents, which caused conformational changes that may have led to enzyme deactivation. Moreover, hydrophobic organic solvents have a dramatic effect on enzyme properties. At lower water content, the enzymes became too dry; thus, they lost their flexibility, resulting in inefficient catalysis [45]. Water acting as a lubricant promotes conformational mobility required for optimal catalysis. It was in concurrence with previous studies whereby the flexibility of lipase B from *Candida antarctica* was reduced when treated with high log *p* values of organic solvents [46]. The flexibility of subtilisin from *Bacillus licheniformis* was also found to be lower in octane (log *p* = 4.183) as compared to in acetonitrile (log *p* = −0.334) [47]. Nevertheless, in this study, there were still *Mp*CAR activities observed (~20–60% relative activities) when it was treated with hydrophobic solvents such as chloroform, octanol, and xylene. Most likely, the support Seplite LX120 was able to protect *Mp*CAR from environmental changes when exposed to the solvents.

The free *Mp*CAR was found to possess higher relative activity after being exposed to hydrophilic and polar organic solvents as compared to the immobilized *Mp*CAR. This phenomenon is probably due to the conformation of the enzyme during its free form, which seems to enhance the enzyme’s ability to catalyze higher substrate conversion in the presence of organic solvents. Even though the immobilized enzyme has lower relative activity when treated with 25% (*v/v*) of hydrophilic solvents, the effect was not significantly detrimental. The support used in this study may not help maintain the enhanced relative activity of *Mp*CAR when treated with organic solvents, as obtained by the free *Mp*CAR. However, compared to the control, the hydrophilic solvents did not inhibit the activity of the immobilized enzyme, as more than 100% of the relative activity of *Mp*CAR was still observed in dimethyl sulfoxide (DMSO), methanol, ethanol, 1-propanol, and butanol. This immobilization strategy could stabilize *Mp*CAR towards organic solvents by preventing the enzyme from unfolding and malfunctioning at its active site caused by solvent penetration [48].

### 3.5. Morphology Analysis Using SEM

**Figure 6:**
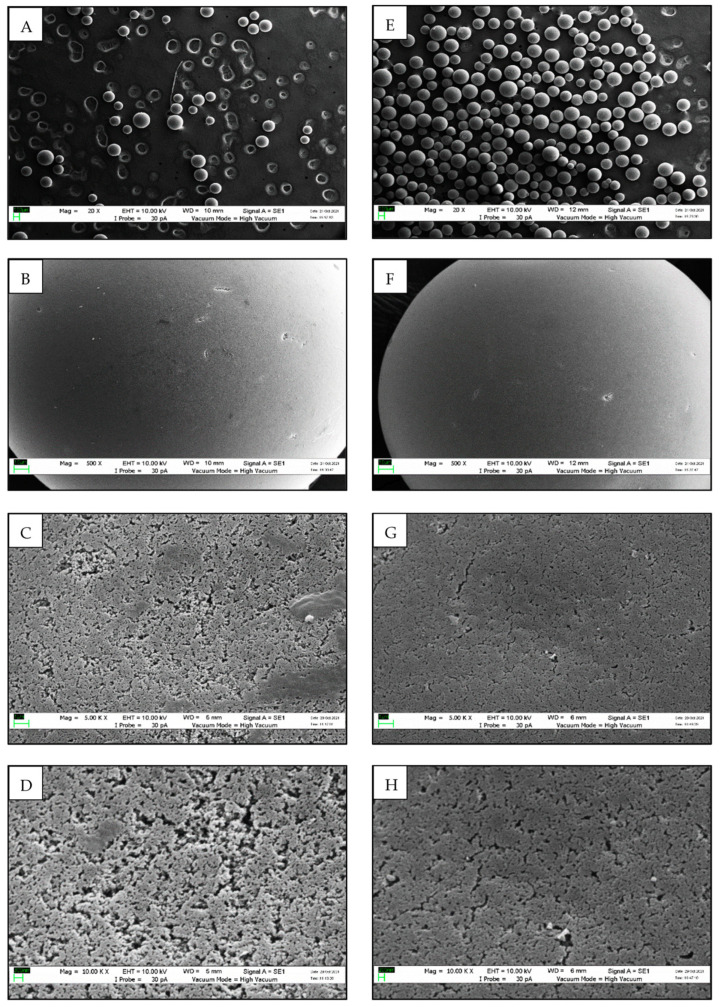
Scanning Electron Microscopy (SEM) images of empty Seplite LX120 and immobilized carboxylic acid reductase from *Mycobacterium phlei* (*Mp*CAR) at 20×, 500×, 5000×, and 10,000× magnifications. Images (**A**–**D**) refer to empty Seplite LX120. Images (**E**–**H**) refer to immobilized *Mp*CAR on Seplite LX120.

SEM micrographs (Figure 6A–D) revealed the morphology of the empty Seplite LX120 support to have a non-porous structure at 20×, 500×, 5000×, and 10,000× magnifications. For comparison, the morphology of immobilized *Mp*CAR was also observed at the same magnifications (Figure 6E–H). At 20× magnification, it was observed that Seplite LX120 is a non-porous spherical in shape support (Figure 6A,B). After immobilizing *Mp*CAR on the support, the void and crack areas became less obvious and most likely to be filled up and covered by the adsorbed *Mp*CAR (Figure 6G,H). As seen in Figure 6G,H, the support with adsorbed enzyme shows a compact and continuous structure after the enzyme immobilization, as compared with the structure of an empty support at 5000× and 10,000× magnifications. Similar SEM images were observed when UDP-glucosyltransferase and sucrose synthase were co-immobilized onto a heterofunctional resin [49]. The decrease in surface roughness and visibility of the spatial position of the support, as shown by SEM images, verified the successful enzyme immobilization [50].

### 3.6. Surface Area Analysis Using BET

BET analysis revealed that the surface area decreased from 122.3347 m^2^/g before *Mp*CAR immobilization to 108.5485 mg^2^/g after immobilization. There was also a slight decrease in the adsorption and desorption pore diameter of the support after the immobilization process (from 0.734 to 0.733 nm and from 7.286 to 7.226 nm of adsorption and desorption pore diameter, respectively). Reduction of the pore volume of Seplite LX120 support was also observed after immobilization (from 0.022 to 0.020 cm^3^/g and from 0.223 to 0.196 cm^3^/g of adsorption and desorption pore volume, respectively). The reduction in surface area, pore diameter, and pore volume indicated that the *Mp*CAR enzyme was successfully adsorbed onto the support. Similar BET analysis results were obtained when lipase and laccase were immobilized onto chicken eggshells and Fe_2_O_3_ yolk-shell particles, respectively [18,51]. It is proven that upon immobilization, *Mp*CAR filled up some of the spaces of the pores on the immobilization support, resulting in a decrease in surface area, pore diameter, and pore volume.

**Figure 7:**
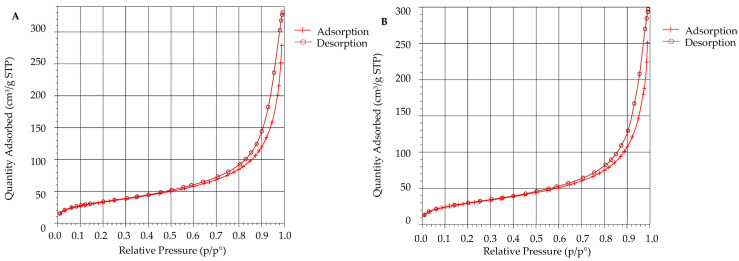
Nitrogen adsorption-desorption isotherm plots of Seplite LX120, before (**A**) and after (**B**) immobilization of carboxylic acid reductase from *Mycobacterium phlei* (*Mp*CAR).

Figure 7A,B show the N_2_ adsorption-desorption isotherm test results to further describe the support capacity or affinity towards *Mp*CAR. Based on IUPAC recommendations, the adsorption isotherms before and after immobilization were categorized as Type II isotherms, and were commonly obtained when the adsorption took place on nonporous or macroporous materials (Figure 7A,B) [52]. By comparing Figure 7A,B, the quantity of nitrogen adsorbed slightly decreased after *Mp*CAR immobilization, implying that there were fewer pores available after immobilization as most of the pores were occupied by the carboxylic acid reductase [53].

### 3.7. Structural Analysis Using FTIR

**Figure 8:**
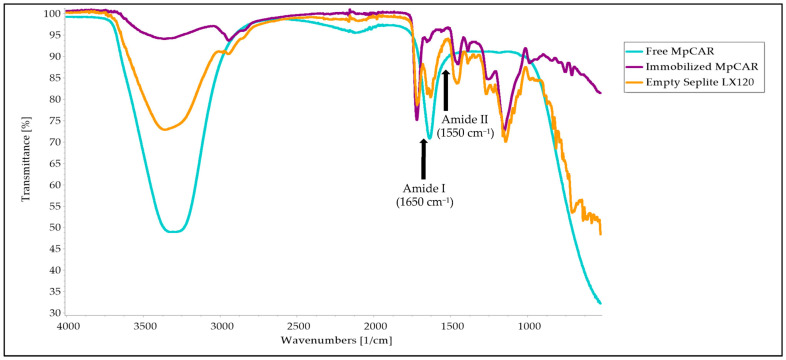
Overlaid Fourier-Transform Infrared Spectroscopy (FTIR) profiles of free carboxylic acid reductase from *Mycobacterium phlei* (*Mp*CAR), immobilized *Mp*CAR onto Seplite LX120, and empty Seplite LX120. Blue line: Free *Mp*CAR; Purple line: Immobilized *Mp*CAR; Orange line: Empty Seplite LX120.

FTIR spectroscopy analysis was used to investigate the bonds involved in the attachment of *Mp*CAR onto the support. Figure 8 shows the overlaid FTIR profiles for all samples, which exhibited similar peak spectra with slight differences. The overlapped OH and NH groups’ strong vibrations of empty Seplite LX120 and free *Mp*CAR can be seen at wavenumbers ranging from 3200 to 3400 cm^−1^. The peak of free *Mp*CAR may be more obvious compared to the peak of immobilized *Mp*CAR at this same wavenumber range, most likely due to the presence of water molecules when the enzyme is in its free form (in liquid form) as compared to when the *Mp*CAR was immobilized onto the support and dried [54]. Since this FTIR spectroscopy analysis was conducted without the standard calibration curve of known concentrations, the results obtained in this section remain qualitative [55]. The peaks of amine stretch detected in this region were sufficient to confirm the presence of the enzyme and the amine functional group of the support in all samples. The bands correlated with protein conformations were detected in the range of 1500–1800 cm^−1^ wavenumbers for all samples, including the empty support since the support is composed of an amine functional group. The changes and shift of peaks (Figure 8) within this range of wavenumbers, including N-H bending vibrations (Amide II at 1550 cm^−1^) and C=O stretching (Amide I at 1650 cm^−1^), indicate that there were alterations in protein secondary structures [50]. These changes indicated that the adsorption of *Mp*CAR onto Seplite LX120 successfully occurred, as similarly discovered when Amano lipase A was immobilized onto a silica matrix [51]. FTIR was also used to examine the changes in the secondary structures of lipase from *Rhizimucor miehei* immobilized onto chitosan as part of the characterization study [56]. In addition, amide bands detected here clearly indicated the preserved enzyme activity after immobilization [57].

### 3.8. Storage Stability and Reusability

**Figure 9:**
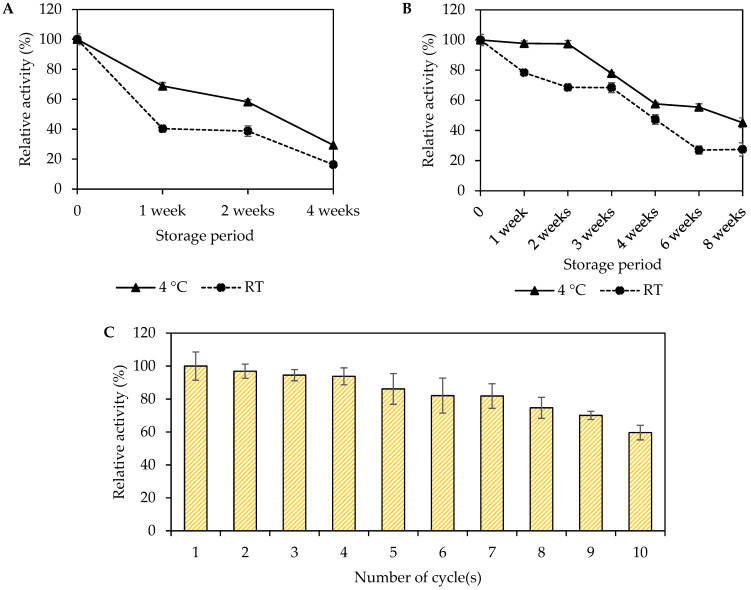
(**A**,**B**) Storage stabilities of free and immobilized *Mycobacterium phlei* carboxylic acid reductase (*Mp*CAR) on Seplite LX120 with optimized immobilization conditions, and (**C**) reusability of immobilized *Mp*CAR. Both free (**A**) and immobilized (**B**) MpCAR were stored for a period of time at 4 and 25 °C (room temperature). The relative activity (%) at day 0 of storage was referred to as 100%. For (**C**), the initial activity of the immobilized *Mp*CAR was taken as 100%. Samples were measured in triplicates.

The stability of the biocatalyst was performed by conducting a series of activity assays for two months of storage for immobilized *Mp*CAR, while for free *Mp*CAR, it was conducted for one month. Storage stability is important for long-term usage, especially in large-scale applications [58]. The long-term storage of immobilized enzymes may contribute to better practicality and cost-effectiveness of the enzymatic process. Upon the immobilization process, the interaction between the support and the enzyme is more robust, which, in this case, may improve the attachment of the carboxylic acid reductase molecule, thus resulting in better storage stability. The storage stability of the immobilized *Mp*CAR was better than the free *Mp*CAR at 4 °C and 25 °C (room temperature) (Figure 9A,B). The immobilized *Mp*CAR retained 45.13% of its initial activity after 8 weeks at 4 °C and 27.41% after 8 weeks at 25 °C. The relative activity of free *Mp*CAR drastically dropped to 29% after 4 weeks at 4 °C and 16.33% after 4 weeks of storage at 25 °C. The decrease in free *Mp*CAR enzyme activity could be attributed to enzyme denaturation caused by the conditions and storage period [59]. As for the immobilized *Mp*CAR, the aggregation or unfolding process of the enzyme would be less likely to occur as the enzymes were well attached to the support. Other immobilized oxidoreductases also showed better storage stability than their free form after being immobilized [60].

The reusability of the immobilized *Mp*CAR is presented in Figure 9C. The reusability assay can be considered as one of the parameters that would be beneficial for industrial applications. Increased reusability can lower production costs by reducing the amount of free CAR in industrial production. Though immobilization conditions have been optimized, enzyme leaching or inactivation may impede the operational stability and repeated use of the immobilized enzyme [61]. In this study, the immobilized *Mp*CAR was repeatedly assayed for several cycles to measure its reusability. It was observed that the immobilized *Mp*CAR retained 59.68% of its catalytic activity even after 10 times of usage. Likewise, immobilization of CAR from *Pycnoporus cinnabarinus* (*Pc*CAR2) showed good reusability as it retained >80% of its initial activity after six cycles of activity assay [30]. Based on the Bradford assay conducted in this study, *Mp*CAR was not detected in the supernatant of the reaction mixture even after 10 cycles of the assay (data not shown). This shows that enzyme leaching was not one of the factors for residual activity reduction of immobilized *Mp*CAR after being reused repeatedly. The presence of an amine (-NH_2_) functional group in the polymer backbone (support) could facilitate strong interactions via hydrophilic-hydrophilic interaction or ionic bonding with the enzyme [62]. The decline in residual activity toward consecutive cycles might be associated with the partial inactivation of the enzyme [63]. Nevertheless, the high reusability of immobilized *Mp*CAR still provides a strong reason for its application in industries.

### 3.9. Bioconversion Using Immobilized MpCAR

**Figure 10:**
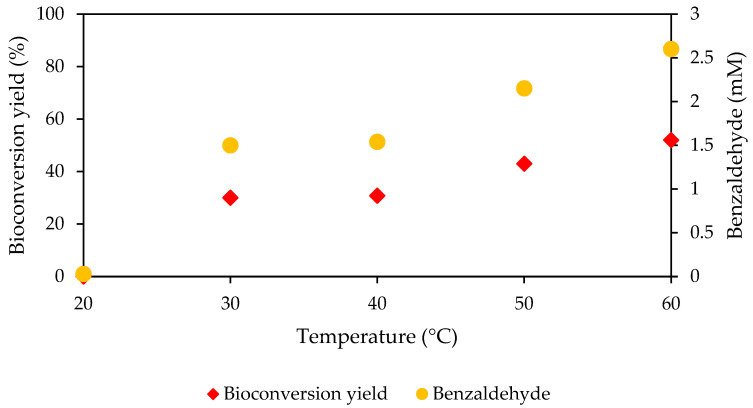
Bioconversion of immobilized carboxylic acid reductase from *Mycobacterium phlei* (*Mp*CAR) onto Seplite LX120. The immobilized *Mp*CAR was incubated with other assay components for 1 h at temperatures ranging from 20 to 60 °C prior to product analysis using HPLC-UV. The substrate concentration supplied was 5 mM. The bioconversion yield (%) and benzaldehyde (mM) concentration were calculated.

Besides the biochemical and biophysical properties of the immobilized enzyme, the ability of the immobilized enzyme to convert substrate to the desired product is also an important parameter that needs to be assessed. Hence, in this study, the preliminary experiment on the bioconversion of benzoic acid to benzaldehyde using immobilized *Mp*CAR was evaluated at different incubation temperatures ranging from 20 to 60 °C, and the benzaldehyde product was quantified using HPLC-UV. These temperatures were chosen since this *Mp*CAR was previously known as a moderately thermostable enzyme [26]. Plus, benzoic acid was selected as the substrate since it is a standard substrate for most carboxylic acid reductases. Based on Figure 10, the bioconversion yield, and the benzaldehyde concentration increased gradually from 20 to 60 °C. The temperature increment causes the molecular movement rate to increase; hence, the reaction rate also increases [64]. The immobilized *Mp*CAR achieved the highest conversion of benzoic acid at 60 °C. The bioconversion yield of 52% at 60 °C, equivalent to 2.6 mM of benzaldehyde, indicated that immobilized *Mp*CAR preferred a higher temperature for its catalytic activity. Almost no benzaldehyde was detected at lower temperatures, such as at 20 °C. A previous study revealed that ~50% of bioconversion of 5 mM of benzoic acid was achieved by immobilized CAR from *Segniliparus rugosus* (*Sr*CAR) after 18 h of incubation at 30 °C [29]. Another finding showed that ~80% bioconversion yield was achieved by immobilized *Pc*CAR, given 2 mM of benzoic acid was supplied for 1 h of reaction incubation at 25 °C [30]. Here, the 2.6 mM of benzaldehyde was quantified after 1 h of incubation of immobilized *Mp*CAR at 60 °C, even though the yield was only about 50%. These findings agree with the trend of optimal activity based on the NADPH consumption assay of immobilized *Mp*CAR, as discussed in Section 3.2 (Figure 3A). Conclusively, at a moderately higher temperature, a greater amount of substrate can be converted to a product with an active immobilized *Mp*CAR. This study demonstrated that the adsorption of *Mp*CAR onto the polymeric support Seplite LX120 was a good approach for maintaining the thermostability of the enzyme. This preliminary bioconversion study of immobilized *Mp*CAR should be a starting point for further exploration of a better bioconversion by the immobilized enzyme, which may include various other carboxylic acid substrates.

## 4. Conclusions

For the first time, the immobilization of *Mp*CAR onto a commercial support, Seplite LX120, was successfully conducted via adsorption. The immobilized *Mp*CAR showed improved biochemical properties, as the enzyme retained its activity over broad temperature and pH ranges. Both free and immobilized *Mp*CAR were stable when treated with 25% (*v/v*) polar organic solvents. The immobilized enzyme was found to be able to be stored longer at 4 and 25 °C compared to the free enzyme. Via immobilization of *Mp*CAR onto Seplite LX120, the operational stability of the enzyme may reach up to 10 cycles. The morphological characterization using SEM showed that the enzyme was successfully adsorbed onto the Seplite LX120 as the cracks and void areas on the support were covered after the immobilization process, as seen in the 10,000× magnification images. Based on the structural characterization using FTIR analysis, there were changes in the secondary structures of the protein after being immobilized onto Seplite LX120 as detected between 1500–1800 cm^−1^ wavenumbers. Nevertheless, the FTIR analysis also confirmed that the activity of the *Mp*CAR was still preserved after immobilization. The BET analysis supported that the immobilization of *Mp*CAR by adsorption technique was successful since the surface area, pore volume, and pore diameter of Seplite LX120 decreased after the immobilization process. Interestingly, HPLC-UV analysis proved that the immobilized *Mp*CAR was active and could convert benzoic acid to benzaldehyde at a higher temperature, at least up to 60 °C of incubation temperature. Overall, the *Mp*CAR immobilized onto Seplite LX120 could be one of the promising biocatalysts for aldehyde production in the flavor and fragrance industries, dependent on its significant improvement in properties, as discussed above.

## Data Availability

Not applicable.
